# Pathological responses to low-dose irradiation and Pepleomycin in Oral squamous cell carcinoma are predictive of Locoregional control

**DOI:** 10.1186/s12885-020-07707-2

**Published:** 2020-12-10

**Authors:** Tomohiro Katagiri, Yoshio Ohyama, Hideo Miyamoto, Yuki Egawa, Toshiaki Moriki, Kazuki Hasegawa

**Affiliations:** 1 Department of Radiation Oncology, Shizuoka City Shizuoka Hospital, 10-93, Ohtemachi, Shizuoka, Aoi-ku 420-8630 Japan; 2 Department of Oral and Maxillofacial Surgery, Shizuoka City Shizuoka Hospital, Shizuoka, Japan; 3 Department of Diagnostic Pathology, Shizuoka City Shizuoka Hospital, Shizuoka, Japan

**Keywords:** Oral cancer, Preoperative chemoradiotherapy, Pathological response

## Abstract

**Background:**

The prognosis of advanced oral cancer remains dismal. While multimodal therapy is beneficial, maintaining the quality of life of long-term survivors is important. Therefore, risk-adapted treatment regimens need to be designed. We herein investigated whether pathological responses in oral cancer patients treated with preoperative chemoradiotherapy predict locoregional recurrence.

**Methods:**

We retrospectively reviewed the data of 51 oral cancer patients who received preoperative radiotherapy and concurrent pepleomycin, followed by curative surgery at our institution between January 2009 and June 2018. Each patient received preoperative external beam irradiation to the primary tumor and lymphatics (2 Gy per day for approximately 3 weeks) concurrent with pepleomycin (2.5 mg/day). Surgery was performed approximately 3–4 weeks after the completion of preoperative chemoradiotherapy. Pathological responses were defined based on the grading system of Oboshi and Shimosato.

**Results:**

Eight, 22, 16, and 5 patients had Oboshi and Shimosato grades 2a, 2b, 3, and 4, respectively. Favorable pathological responses (grades 3 and 4) were observed in 41.2% of patients (21 out of 51 patients). The pathological response and number of pathological lymph node metastases were identified as significant prognostic factors for locoregional control in the univariate analysis. Three-year locoregional control rates were 100 and 56.6% in patients with favorable and unfavorable pathological responses, respectively.

**Conclusions:**

The present study demonstrated that pathological tumor responses to preoperative chemoradiotherapy are a useful predictive factor for locoregional control.

## Background

The prognosis of advanced oral squamous cell carcinoma (OSCC) remains dismal, but may be improved by multimodal therapy. Advanced OSCC is typically treated by surgery with or without postoperative radiotherapy or chemoradiotherapy based on pathological findings [1]. Preoperative chemoradiotherapy has been conducted in some centers, and a literature-based meta-analysis indicated no significant differences in survival between pre- and postoperative adjuvant radiotherapy; however, data from prospective randomized studies comparing these two therapies for OSCC remain limited [2]. The Japanese oral cancer guidelines state that preoperative chemoradiotherapy may be beneficial for locoregional control and overall survival (OS), and increase the potential for limited surgery on primary sites in patients with resectable locally advanced OSCC [3].

Since the treatment outcomes of head and neck cancer are improved by multidisciplinary therapy, efforts to maintain the quality of life (QOL) of long-term survivors are crucial. Adjuvant radiotherapy after surgery for head and neck cancer is associated with late adverse events, including xerostomia and dysphagia, which deteriorate QOL [4]. Lower-dose irradiation to the parotid gland and swallowing structures is associated with fewer and less severe late adverse events, resulting in better QOL [5, 6]. However, less intensive therapy, such as lower-dose irradiation, may increase the risk of recurrence, and oral cancer patients with recurrence, regardless of an initially early stage, have a poor prognosis [7]. Therefore, risk-adapted treatment regimens need to be designed. The difficulties associated with the selection of initial treatment strategies, particularly adjuvant radiotherapy and less or more intensive treatments, are attributed to the challenges of maintaining the balance between the preservation of function and cosmesis and locoregional control in head and neck cancer. Therefore, the identification of prognostic factors is crucial for predicting the prognosis of each patient and selecting appropriate optimal therapy.

We performed preoperative chemoradiotherapy consisting of low-dose irradiation and pepleomycin followed by surgery for oral cancer patients with primary tumors of 3 cm or larger or clinically positive lymph node (LN) metastases in an attempt to improve curability and enable less invasive surgery. In the present study, we investigated whether pathological responses in oral cancer patients treated with preoperative chemoradiotherapy predict locoregional recurrence.

## Methods

### Patient eligibility

Between January 2009 and June 2018, 89 patients with oral cancer were referred to the Department of Radiation Oncology, Shizuoka City Shizuoka Hospital. Among these patients, 7 who received palliative radiotherapy, 6 curative radiotherapy without surgery for either primary or recurrent disease, 14 postoperative radiotherapy with or without preoperative radiotherapy, and 10 preoperative radiotherapy and concurrent systematic therapy other than pepleomycin were excluded. The remaining 51 patients treated with preoperative radiotherapy and concurrent pepleomycin followed by curative surgery were examined in the present study.

The pretreatment work-up included a physical examination, contrast-enhanced computed tomography (CECT) unless contraindicated and/or magnetic resonance imaging (MRI) to assess the tumor extent, and/or 2-deoxy-2[F-18] fluoro-D-glucose positron emission tomography (FDG-PET) to detect LN metastases and distant metastases. Patient staging was revised according to the 7th UICC clinical staging system. All tumors were diagnosed as squamous cell carcinoma by a histopathological examination of biopsy specimens. This retrospective study was approved by the Institutional Review Board, and written informed consent was waived because of its retrospective design.

### Treatment

Radiotherapy was performed using EXL-15SP (Mitsubishi) with energy of 6MVX before July 2016 and with Clinac iX (Varian) with energy of 4 or 10 MVX after July 2016. Patients were treated with two- or three-dimensional conformal radiotherapy. Each patient received external beam irradiation to the primary tumor and lymphatics (2 Gy per day for approximately 3 weeks). The total dose was selected based on the degree of acute mucositis and extent of shrinkage of the primary tumor at the discretion of the treating physician. The elective nodal field was mainly decided based on the primary site and extent of disease. Concurrent chemotherapy with low-dose pepleomycin (2.5 mg/day) was administered subcutaneously in a 10-h continuous injection daily to prevent interstitial pneumonia during radiation [8]. Surgery was performed approximately 3 weeks after the completion of preoperative chemoradiotherapy. Primary tumor surgery with or without neck dissection was conducted. Limited surgery was performed on patients with a favorable clinical response in soft tissue organs, such as the tongue and buccal mucosa. Neck dissection was conducted based on the risk of nodal spread and discretion of the surgeon. Comprehensive or selective neck dissection was performed depending on the extent of the primary tumor, primary tumor thickness, number and site of clinical lymph node metastasis, and presence/absence of extranodal extension. In reconstruction, a radial forearm flap, rectus abdominis flap, scapula osteocutaneous flap, and latissimus dorsi flap were used where necessary. The safety margins of the primary lesion were 10–20 mm from palpable margins. Frozen sections obtained during surgery were examined to confirm adequate margins.

### Histopathological evaluation

All tumor specimens were fixed in 10% buffered formalin, cut into 5-mm-thick sections, and then embedded in paraffin. We took haematoxylin and eosin-stained slides of all tumor sections and evaluated histopathological response by all slides (the median number of slides were 8, range: 1–23). Pathological responses were defined based on the grading system of Oboshi and Shimosato [9, 10]. Grade 0 corresponds to no response. In grade 1 corresponding to a minor response, cancer cells are damaged without the destruction of the tumor structure. In grades 2a and 2b, destruction of the tumor structure is observed in addition to damaged cancer cells (in less than 75% of the analyzed tissue in grade 2a and more than 75% in grade 2b). In grade 3, non-viable cancer cells are present. In grade 4, no cancer cells are found. A favorable pathological response is defined as the absence of any viable tumor cells (grades 3 and 4), equivalent to a complete response. An unfavorable pathological response was defined as the presence of any viable tumor cells (grades 0-2b). Two experienced pathologists (Y.E. and T. M, our co-authors) performed pathological evaluation of the tumors. Disagreement about pathological response between the two pathologists were resolved by consensus.

### Follow-up

After the completion of surgery, patients were followed up every month by a clinical examination and every 3 months by computed tomography during the first 2 years, and every 6 months thereafter until death or data censoring. Clinical data on adverse events were retrospectively collected from medical records and the grade was determined according to National Cancer Institute Common Toxicity Criteria for Adverse Events (CTCAE) version 4.0.

### Outcomes and statistical analysis

OS and disease-specific survival (DSS) were calculated from the date of the start of chemoradiotherapy to the date of death from any cause or from oral cancer, respectively. Time to locoregional recurrence was calculated from the date of the start of chemoradiotherapy to the date of first local or regional recurrence, respectively.

A survival analysis was conducted using the Kaplan-Meier method and Log-rank test. Statistical analyses were performed with Fisher’s exact test for categorical variables or the Wilcoxon rank-sum test for numerical variables. All tests were two-sided at a significance level of 0.05. Analyses were performed using R version 3.5.2 (The R Foundation for Statistical Computing, Vienna, Austria).

## Results

### Patients

Baseline clinical and treatment characteristics are shown in Table 1. Median age was 68 years old (31–84). Ten patients had cervical LN metastases after the initial treatment. Clinical stage T3–4 primary tumors and clinical stage N2 LN metastases were observed in 49.0 and 45.1% of patients, respectively. Radiotherapy was targeted to the unilateral neck in 23.5% of patients and the whole neck in 76.5%. The median total dose of radiotherapy was 30 Gy (22–36 Gy). Surgery was performed within 4 weeks of preoperative chemoradiotherapy (median, 21 days) in 94.1% of patients (48 out of 51). Ipsilateral and bilateral neck dissection were performed on 56.9 and 31.4% of patients, respectively.

**Table 1:** Clinical and treatment characteristics

	*n* = 51
Sex	
Male	27
Female	24
Age	
Median	68
Range	31–84
Disease	
Primary	38
Recurrent	13
Sites	
Tongue	9
Maxillary gingiva	6
Mandibular gingiva	17
Buccal mucosa	2
Soft palate	1
Floor of the mouth	6
Occult cervical lymph node metastasis	10
Clinical T classification	
Tx	10
T1	0
T2	16
T3	9
T4	16
Clinical N classification	
N0	13
N1	15
N2a	2
N2b	17
N2c	4
N3	0
Treatment machine	
EXL-15SP	43
Clinac iX	8
Radiation Field	
Hemineck	12
Whole neck	39
Total dose	
Median (range)	30 (22–36) Gy
< 30 Gy	20
≥ 30 Gy	31
Neck dissection	
None	6
Ipsilateral neck	29
Bilateral neck	16

### Pathological responses to chemoradiotherapy

Pathological stages and tumor characteristics are shown in Table 2. No patients showed grade 0–1 responses of the Oboshi and Shimosato classification, while grades 2a, 2b, 3, and, 4 were observed in 8, 22, 16, and 5 patients, respectively. A favorable pathological response (Oboshi and Shimosato grades 3 and 4) was noted in 41.2% of patients (21 out of 51 patients).

**Table 2:** Pathological characteristics at surgical resection

Number of pathological lymph node metastases	
0–1	36
≥ 2	15
Site of pathological lymph node metastases	
Level IV or V	3
Other level or no metastasis	48
Pathological response based on the Oboshi and Shimosato classification	
Grade 0–1	0
Grade 2a	8
Grade 2b	22
Grade 3	16
Grade 4	5

### Locoregional control and OS

**Fig. 1:**
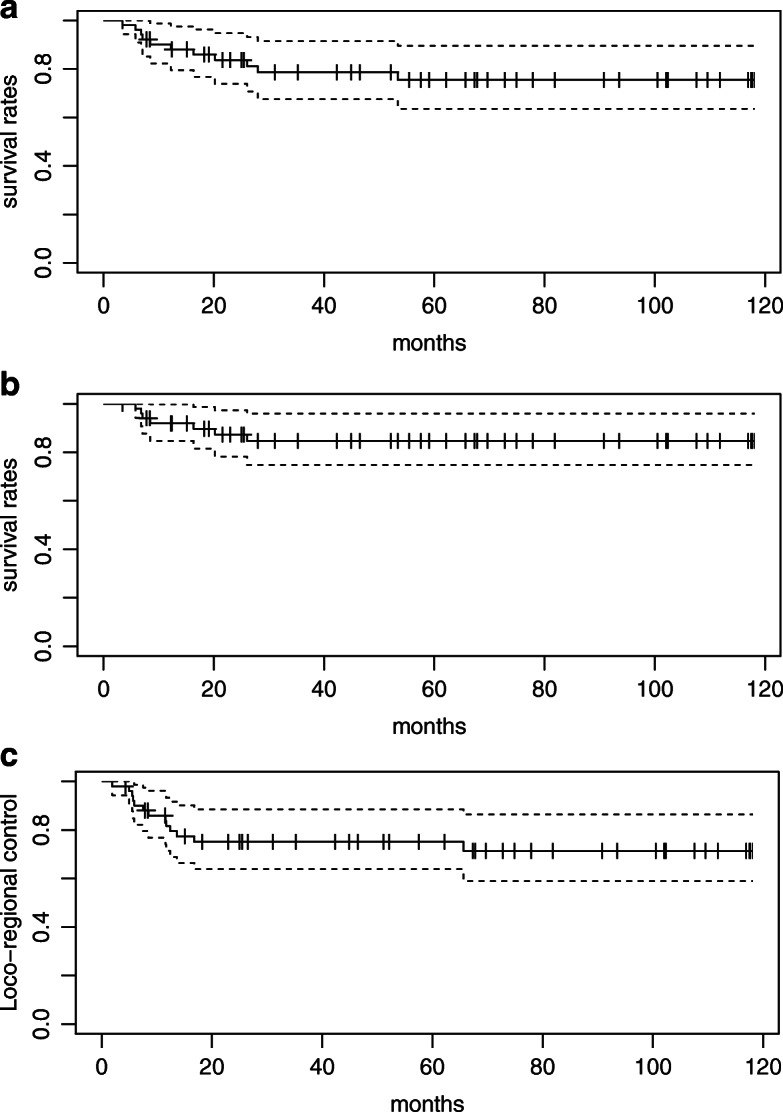
Overall survival rates (**a**), disease-specific survival rates (**b**), and locoregional control rates (**c**)

**Fig. 2:**
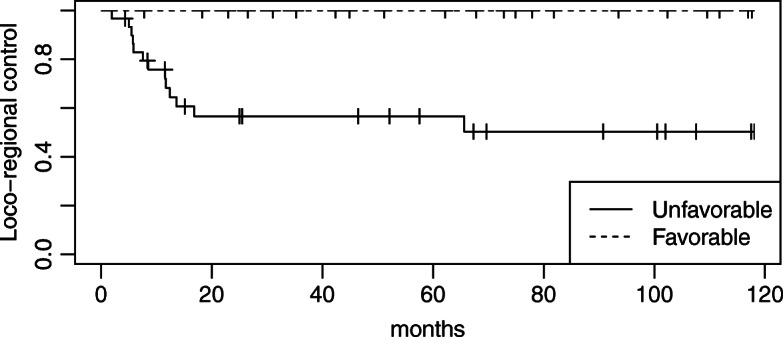
Locoregional control rates in patients with favorable (Grades 3, 4) versus unfavorable (Grades 2a, 2b) pathological responses

**Fig. 3:**
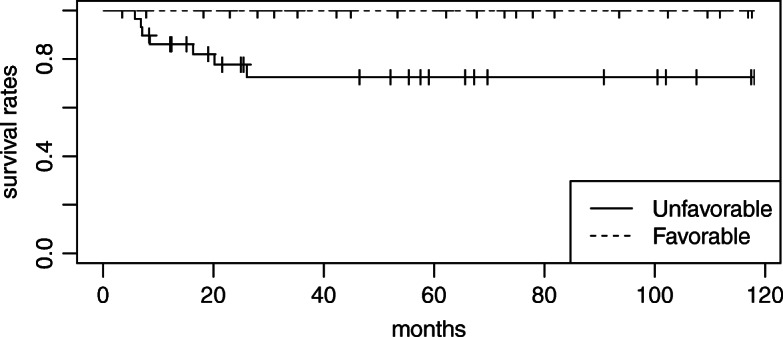
Disease-specific survival rates in patients with favorable versus unfavorable pathological responses

**Fig. 4:**
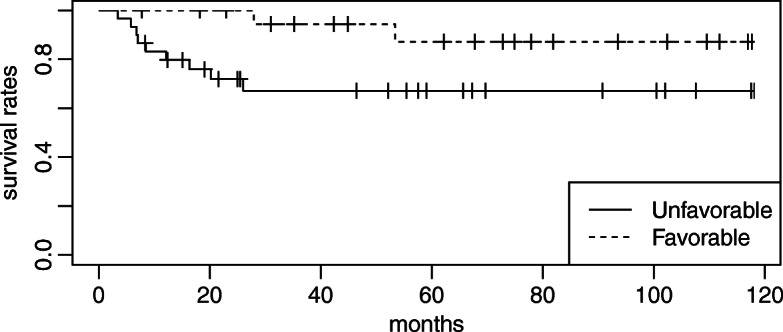
Overall survival rates in patients with favorable versus unfavorable pathological responses

The median follow-up period was 52.1 months. At the end of the follow-up, 13 patients had disease progression and 11 had died. The cause of death was disease progression in 8 patients, sepsis due to other diseases in 2, and colon cancer in 1. The sites of failure were locoregional recurrence in 13 patients and distant recurrence in 6. All locoregional recurrence occurred as the first site of recurrence. Three-year OS, 3-year DSS, and 3-year locoregional control rates in all patients were 78.6, 84.7, and 75.1%, respectively (Fig. 1). Besides the number of pathological LN metastases, the pathological response was identified as the only significant prognostic factor for locoregional control in the univariate analysis (Table 3). Three-year locoregional control rates were 100 and 56.6% in patients with favorable and unfavorable pathological responses, respectively. The locoregional control rate was significantly higher for patients with favorable pathological responses than for those with unfavorable responses (*p* < 0.05; Fig. 2). A favorable pathological response correlated with DSS (*p* < 0.05; Fig. 3) and was associated with OS (*p* = 0.05; Fig. 4). No other relationships were observed between pathological responses and other clinicopathological factors, including the number of pathological LN metastases or treatment-related factors (Table 4). In 38 cases excluding recurrent disease, 3-year locoregional control rates were 100 and 62.9% in patients with favorable and unfavorable pathological responses, respectively (*p* < 0.05). Similar trends were observed for OS and DSS (*p* < 0.05). In 25 cases of clinically advanced-T-stage (T3–4) disease, 3-year OS, 3-year DSS, and 3-year locoregional control rates were 77.3, 83.2, and 69.1%, respectively. The locoregional control rate was significantly higher for advanced-T-stage patients with favorable pathological responses than for those with unfavorable responses (100% compared with 42.7%; *p* = 0.004). A favorable pathological response showed marginally significant and no association with DSS and OS (*p* = 0.06 and *p* = 0.2), respectively.

**Table 3:** Log-rank analysis of locoregional control

	n	3-year locoregional control	*p*-value
Disease			
Primary	38	80.3%	*p* = 0.2
Recurrent	13	61.5%	
Number of pathological LN metastases			
0	23	90.2%	*p* < 0.05
≥ 1	28	63.4%	
≤ 1	36	82.1%	*p* = 0.1
≥ 2	15	59.3%	
Treatment machine			
EXL-15SP	43	75.8%	*p* = 0.7
Clinac iX	8	75.0%	
Total dose			
< 30 Gy	20	77.9%	*p* = 0.9
≥ 30 Gy	31	73.2%	
Radiation field			
Unilateral neck	12	75.0%	*p* = 1.0
Whole neck	39	75.4%	
Neck dissection			
None/Ipsilateral	35	81.7%	*p* = 0.1
Bilateral	16	61.4%	
Pathological response based on the Oboshi Shimosato classification			
≥ Grade 3	21	100%	*p* < 0.001
≤ Grade 2	30	56.6%

**Table 4:** Comparison of clinicopathological factors between favorable and unfavorable pathological groups

	≤Grade 2 *n* = 30	≥Grade 3 *n* = 21	
Disease			
Primary	21	17	*p* = 0.518
Recurrent	9	4	
Clinical T classification			
cTX-2	16	10	*p* = 0.7793
cT3–4	14	11	
Clinical N classification			
cN0–1	18	10	*p* = 0.4078
cN2a-	12	11	
Number of pathological LN metastases			
0	13	10	*p* = 0.7828
≥ 1	17	11	
≤ 1	23	13	*p* = 0.3514
≥ 2	7	8	
Median (range)	1 (0–10)	1 (0–5)	*p* = 0.903
Total dose median (range)	30 (25–36) Gy	30 (22–36) Gy	*p* = 0.7271
Period from radiotherapy end to surgery			
Median (range)	23 (12–33) days	20 (8–36) days	*p* = 0.2051
Treatment machine			
EXL-15SP	25	18	*p* = 1.0
Clinac iX	5	3	
Radiation field			
Unilateral neck	7	5	*p* = 1.0
Whole neck	23	16	
Neck dissection			
None/ipsilateral	18	17	*p* = 0.14
Bilateral	12	4	

### Adverse events

Grade 2/3 anemia, neutropenia, and thrombocytopenia were observed in 14/2, 15/0, and 0/1 patients, respectively. The patient with grade 3 thrombocytopenia had a coexistent alcoholic liver cirrhosis. No patient showed grade 4 myelosuppression. Thirteen and 5 patients showed grade 1 and 2 late xerostomia, respectively. Grade 3 aspiration, aspiration pneumonia requiring admission, was observed in 4 patients.

## Discussion

In the present study, we demonstrated that pathological responses to preoperative chemoradiotherapy predicted locoregional control rates. Although our patients received low-dose irradiation (median 30 Gy, range 22–36 Gy), locoregional recurrence was not detected in those with favorable pathological responses. The present results indicate the potential of a favorable pathological response as a good surrogate marker for controlling microscopic and macroscopic tumors even with low-dose irradiation in combination with chemotherapy and surgery. Low-dose irradiation may result in fewer adverse events and better QOL.

Previous studies reported a relationship between the prognosis of patients with oral or oropharyngeal cancer and responses to preoperative chemoradiotherapy [10, 11]. Patients with stage II-IV oral or oropharyngeal cancer were treated with preoperative chemoradiotherapy (50 Gy in 25 daily fractions and concurrent chemotherapy with mitomycin C and fluorouracil), followed by radical surgery. Two-thirds of patients were responders, who were defined as having no vital tumor or minimal tumor remnants encompassing less than 5%. Locoregional control was significantly better for responders than for non-responders (2 years; 92.4–94.2% vs. 68.0–69.8%, *p* < 0.001) [11]. Kirita et al. reported a relationship between survival and pathological responses after preoperative chemoradiotherapy (40 Gy in 20 daily fractions, cisplatin- or carboplatin-based chemotherapy) in patients with stage II-IV tongue cancer. Responders were defined as grades 2b, 3, and 4 based on the Oboshi/Shimosato classification. Progression-free survival rates were higher for responders than for non-responders (2 years; 95.7–100 vs. 50%). Favorable pathological responses (grades 3/4 in 25/5 patients, respectively) were observed in 30 patients (69.8%) based on our definition [10]. The present study showed a favorable pathological response based on the Oboshi/Shimosato classification in 41.2% of patients, which was lower than that in previous studies. This discrepancy may be attributed to lower-dose preoperative irradiation and differences in chemotherapy regimens, pathological response criteria, and patient backgrounds. However, the prognosis of responders in the present study was similar to that in previous studies, even though our patients received lower-dose irradiation. Therefore, lower-dose irradiation may be sufficient for patients with favorable pathological responses. To the best of our knowledge, few studies have investigated the relationship between the prognosis of patients with oral cancer and responses to preoperative low-dose chemoradiotherapy.

Surgical resection may exert adverse effects on appearance, swallowing, speech, and shoulder function, while the addition of adjuvant radiotherapy may cause xerostomia, altered taste, dental decay, and osteoradionecrosis with deteriorating dysphagia [12]. Combined treatments may provide the best chance of a cure at the cost of more frequent and severe adverse events and lower QOL. A systematic review of 26 retrospective studies on QOL in head and neck cancer patients treated with surgery alone or in combination with adjuvant radiotherapy demonstrated that the addition of adjuvant radiation worsened mouth dryness, thick saliva, and difficulty with mouth opening [4]. The current National Comprehensive Cancer Network guidelines recommend adjuvant radiotherapy of at least 44 and 60 Gy at 2 Gy/fraction to lower and higher risk regions of the neck, respectively [1]. Radiation dose-response relationships were previously reported between swallowing structures and late dysphagia [13] and the parotid glands and late xerostomia [5, 14]. Lee et al. demonstrated that the threshold value of the parotid mean dose for the incidence of LENT-SOMA grade 3 or higher xerostomia was 20 Gy [5]; however, there was no threshold dose for RTOG/EORTC grade 4 xerostomia in the study by Dijkema et al. [14]. Severe late xerostomia was observed in 50% of patients with parotid mean doses of 39.9–43.6 Gy [5, 14]. A QUANTEC review recommended that the mean dose to each parotid gland needs to be kept as low as possible, consistent with desired clinical target volume coverage [15]. Levendag et al. reported that the threshold value in the superior constrictor muscle, one of the swallowing structures, for the incidence of RTOG grade 3 or more dysphagia was 21 Gy [13]. Intensity-modulated radiation therapy (IMRT) is a useful technique for delivering a high dose to the tumor and minimizing the dose to organs at risk (OAR) by varying the beam intensity in the radiation field. This technique was previously shown to reduce the incidence and degree of late adverse events, including late xerostomia and dysphagia, without decreasing locoregional tumor control [16, 17]. However, in a situation in which parotid glands and swallowing structures are within or near tumor tissues, IMRT cannot reduce the radiation dose for these OAR sufficiently when standard high-dose radiotherapy consisting of 70 Gy in curative settings or 60 to 66 Gy in adjuvant settings was delivered in 2-Gy fractions. Previous studies demonstrated that the average mean doses to pharyngeal constrictors and the ipsilateral/contralateral parotid gland were 58 and 47.6/25.4 Gy, respectively, in head and neck cancer patients treated with standard curative radiotherapy, even with the IMRT technique [16, 17]. Therefore, lower-dose irradiation consisting of 30 Gy in 2-Gy fractions in the present study is considered to offer the clinically meaningful benefit of alleviating late radiation toxicity. Ongoing clinical trials are investigating whether dose de-escalations may be safely performed without the worsening of treatment outcomes in head and neck cancer patients, mainly human papilloma virus (HPV)-related oropharyngeal cancer patients, in curative or adjuvant radiotherapy settings [18]. Although the prognosis of HPV-related head and neck cancer patients is good [19], the low incidence of HPV-related oral cancer (13.8% by p16 positivity) suggests a minor role in oncogenesis in Japanese oral cancer patients [20]. Although the majority of our patients were considered to have HPV-unrelated oral cancer, preoperative lower-dose radiotherapy with chemotherapy and surgery may be sufficient for tumor control in patients with favorable pathological responses.

The Japanese oral cancer guidelines recommend pre- or postoperative chemoradiotherapy for better locoregional control and OS in patients with advanced oral cancer [3]. The NCCN guidelines recommend postoperative radiotherapy for oral cancer patients with adverse risk features, including extranodal extension, a positive margin, pT3 or T4 primary tumors, N2 or N3 nodal disease, nodal disease at level IV or V, perineural invasion, vascular embolism, and lymphatic invasion, without describing their recommendations on preoperative radiotherapy for oral cancer. Since our patients were treated with preoperative chemoradiotherapy and surgery, the pathological data obtained cannot be compared with those from patients undergoing upfront surgery. In our study, about half of the patients (10 of 21 patients) with clinically more than one positive neck node were downstaged to no or one pathologically positive neck node by preoperative chemoradiotherapy. Systematic reviews on the diagnostic accuracy of oral cancer demonstrated that CT and MRI were useful for evaluating the extension of the primary tumor site, and FDG-PET may contribute to the detection of metastatic cervical lymph nodes [21]. Clinical staging by multi-modality imaging is consistent with pathological staging in oral cancer. The pretreatment work-up in the present study revealed that 76.5% of our patients had clinical T3–4 and/or clinical N2–3 and/or recurrent disease, while the remaining 12 patients had clinical T2N0–1. Occult metastases have been reported in 13–33% of T1 tumors and 37–53% of T2 tumors at the time of diagnosis, even in clinical early T-stage and node-negative oral cancer patients classified by morphological imaging only [22]. Some of our patients with clinical T2N0–1 may have lymph node metastases pathologically and be suitable for postoperative radiotherapy. Therefore, many of our patients receiving preoperative chemoradiotherapy and undergoing surgery may be candidates for upfront surgery and postoperative chemoradiotherapy, although there was a possibility of overtreatment with preoperative chemoradiotherapy in some of our early stage oral cancer patients. The National Cancer Database study showed the patients with clinical T3–4 oral cancer had a 3-year OS of 49.7% when treated with surgery and postoperative radiotherapy compared with 36.0% when treated with definitive chemoradiotherapy [23]. A study by Zhong et al. demonstrated that 2-year OS and 2-year progression-free survival (PFS) were 90.1 and 79.9% in the clinical T3–4 oral cancer patients receiving induction chemotherapy with docetaxel, cisplatin, and fluorouracil followed by surgery and postoperative radiotherapy (about 95% of the patients received at least 54Gy irradiation), respectively [24]. Our clinical T3–4 patients had a 2-year OS and 2-year PFS of 83.2 and 66.3%, respectively. Our treatment outcomes might not be inferior to those in the above-described studies.

The present study has several limitations. The sample size in this single-institution study was small. Furthermore, it was a retrospective study that may have had a selection bias. Several patients with locoregionally advanced disease underwent other treatments, including curative radiotherapy or upfront surgery with postoperative radiotherapy because they were older or had comorbidities. Therefore, the present results cannot be generalized to locoregionally advanced oral cancer patients with an older age and comorbidities. In addition, the definition of favorable pathological response was different among studies. Favorable pathological response was defined as the absence of any viable tumor cells (grades 3 and 4) in our study, whereas it was defined as a few or no viable tumor cells (grades 2b-4) in a study of Kirita et al. [10]. Judging whether viable tumor cells were present (grades 0-2b) or absent (grades 3 and 4) seemed more concordant between observers than calculating the number of viable tumor cells, which was based on the discrimination between grades 2a and 2b. However, the overestimation bias of grade 3 response may exist if a small number of tumor sections for evaluation leads to overlooking viable tumor cells. This bias can be minimized in our study because the number of tumor sections for evaluation was thought to be enough (median 7, range: 1–13). Furthermore, we selected pepleomycin, a bleomycin derivative with less pulmonary toxicity than bleomycin, as the radiation sensitizer because it was expected to be less myelotoxic than platinum-based chemotherapy even though it has been associated with an increased risk of pulmonary fibrosis and more severe mucositis. Concurrent chemoradiotherapy with bleomycin has been administered to patients with head and neck cancer and esophageal cancer [25, 26]. Platinum-based chemoradiotherapy is now the standard treatment regimen for these cancers, partly because of the pulmonary toxicity of bleomycin and clinical evidence supporting the utility of platinum-based chemoradiotherapy in various cancers. None of the patients in the present study developed pulmonary fibrosis, which may have been due to the continuous injection of pepleomycin [8]. Another limitation is that the adverse events of radiotherapy, including dry mouth and dysphagia, were not systematically reported for patients in the present study, although low-dose irradiation was expected to alleviate toxicity. The frequency and severity of adverse events might be underestimated in our study. Prospective QOL survey will be needed to validate our results. Preclinical studies previously demonstrated that the administration of pepleomycin sensitized the effects of radiation on cancer cells, but not on normal oral mucosal or salivary gland cells [27, 28]. The tumor specificity of this drug may enable lower-dose irradiation to control oral cancer with negligible damage to normal tissues. Further studies are needed to confirm these results. In addition, surgical and radiation techniques were non-uniform because they were selected at the discretion of the surgeon and radiation oncologist. However, no significant differences were observed in surgical or radiation techniques between patients with favorable and unfavorable pathological responses.

## Conclusions

The present results demonstrated that pathological tumor responses to preoperative chemoradiotherapy are useful predictive factors for locoregional control and DSS in combination with chemotherapy and surgery. Low-dose irradiation may be sufficient for patients with good pathological responses; therefore, these patients may avoid radiation-induced late toxicity associated with higher doses of irradiation. On the other hand, patients with unfavorable pathological responses need to receive more aggressive treatments, such as more intensive chemotherapy, higher doses of preoperative irradiation, or the addition of adjuvant irradiation.

## Data Availability

The datasets generated during the current study are not publicly available since they will contain patient data and the informed consent agreement does not include sharing data publicly. An anonymized form of the data could be made available from the corresponding author upon reasonable request.

## Abbreviations

OSCC: Oral squamous cell carcinoma
OS: Overall survival
QOL: Quality of life
LN: Lymph node
CECT: Contrast-enhanced computed tomography
MRI: Magnetic resonance imaging
FDG-PET: 2-Deoxy-2[F-18] fluoro-D-glucose positron emission tomography
CTCAE: Common toxicity criteria for adverse events
DSS: Disease-specific survival
IMRT: Intensity-modulated radiation therapy
OAR: Organs at risk
HPV: Human papilloma virus
PFS: Progression-free survival
